# Detecting Mortality Trends in the Netherlands Across 625 Causes of Death

**DOI:** 10.3390/ijerph16214150

**Published:** 2019-10-28

**Authors:** Marianna Mitratza, Anton E. Kunst, Jan W. P. F. Kardaun

**Affiliations:** 1 Department of Public Health, Amsterdam UMC, University of Amsterdam, 1105 AZ Amsterdam, The Netherlandsjwpf.kardaun@cbs.nl (J.W.P.F.K.); 2 Department of Health and Care, Statistics Netherlands, 2090 HA The Hague, The Netherlands

**Keywords:** mortality, monitoring, causes of death, size, ICD-10, long-term trends, short-term fluctuations

## Abstract

Cause of death (COD) data are essential to public health monitoring and policy. This study aims to determine the proportion of CODs, at ICD-10 three-position level, for which a long-term or short-term trend can be identified, and to examine how much the likelihood of identifying trends varies with COD size. We calculated annual age-standardized counts of deaths from Statistics Netherlands for the period 1996–2015 for 625 CODs. We applied linear regression models to estimate long-term trends, and outlier analysis to detect short-term changes. The association of the likelihood of a long-term trend with COD size was analyzed with multinomial logistic regression. No long-term trend could be demonstrated for 216 CODs (34.5%). For the remaining 409 causes, a trend could be detected, following a linear (211, 33.8%), quadratic (126, 20.2%) or cubic model (72, 11.5%). The probability of detecting a long-term trend increased from about 50% at six mean annual deaths, to 65% at 22 deaths and 75% at 60 deaths. An exceptionally high or low number of deaths in a single year was found for 16 CODs. When monitoring long-term mortality trends, one could consider a much broader range of causes of death, including ones with a relatively low number of annual deaths, than commonly used in condensed lists.

## 1. Introduction

Mortality statistics are a key source of information in public health, epidemiology and medicine. As part of the vital statistics registries, they cover entire national populations, extend over long periods of time and are easily accessible. Among others, these statistics are used to monitor cause-specific mortality, in order to identify trends that may inform disease prevention, screening and surveillance.

Both short- and long-term mortality trends can be monitored to identify changes that may prompt health policy actions. Trends in the short term, defined as a maximum period of a year, are particularly important to identify sudden population-level changes and events, such as outbreaks of communicable diseases. Long-term mortality trends, defined as secular changes, as observed across several years, may reflect gradual changes in the incidence or case-fatality of specific diseases or injuries. 

Causes of death (CODs) are—in most countries—classified according to the 10th revision of the International Classification of Diseases (ICD-10) [1]. The ICD-10 contains 1761 codes at a three-position level, which refers to a single condition, a group of diseases, “other” or “unspecified” conditions, and more than 14,000 codes at the four- and five-position level. Studies monitoring trends in mortality by cause of death are often restricted to a few specific causes of interest, or to a systematic, but abbreviated, list of leading CODs [2,3]. 

The large number of ICD-10 codes raises the question of how many of these codes should be distinguished when tabulating, analyzing and publishing mortality trends. This applies especially to national statistical offices, both within Europe and in other continents, as they have to decide on the level of detail to include when publishing annual cause-specific mortality data. Monitoring all available ICD-10 codes, such as the 1761 codes at the three position level, may produce valuable signals for health policy, that would go unnoticed when using abbreviated lists of leading CODs. On the other hand, this approach may also produce uninformative signals, such as large random fluctuations in the annual number of deaths from smaller causes.

The size of a COD—in terms of mean annual number of deaths—may offer a criterion to decide which ICD-10 codes should be distinguished. All else being equal, the likelihood of detecting a trend increases with an increase in COD size. A detailed description of this relationship may, therefore, help develop guidelines for selecting CODs. Such guidelines may be particularly relevant regarding the forthcoming release of the ICD-11 version, which may increase the number of possible COD codes, and consequently decrease the average number of deaths per COD code. The 2018 version of ICD-11 for Mortality and Morbidity Statistics has more than double the number of disease entity codes, compared to ICD-10 [4].

The general aim of this study was to determine the level of detail, in terms of COD size, at which the ICD-10 classification can be best used for presenting mortality trends in national populations. The specific objective of this study was to estimate the proportion of causes of death for which we can observe a long-term trend or a short-term change, and how the probability of finding a long-term trend relates to COD size. To do this, we assessed trends in COD in the Netherlands over a period of 20 years.

## 2. Materials and Methods

We analyzed annual mortality data from Statistics Netherlands [5] for the period 1996–2015, regarding each underlying COD at the ICD-10 three-position level, as reported on the death certificate and coded at Statistics Netherlands. This period was chosen as the ICD-10 was introduced in the Netherlands in 1996, and 2015 was the last year with available data at the time of initiation of this study.

We excluded 1136 CODs with less than three annual deaths on average, because most of these CODs had predominantly zero or only zero annual deaths. No death (only zero values) was observed for 415 CODs throughout the 20-year period, while the rest of the excluded CODs accounted together for 476 deaths per year on average, which corresponded to 0.4% of the total number of deaths.

For the remaining 625 CODs, we calculated age-standardized counts of deaths using age-standardized mortality rates, calculated with the direct method, using the Dutch population in 2005 as a reference population (i.e., mid-period). This method intended to control for annual changes in the age distribution of the population. The population size was 15.5 million persons in 1996, with the age distribution (<20, 20–40, 40–65, 65–80, ≥80 years) being 24%, 32%, 31%, 10% and 3%, respectively. The corresponding numbers for 2015 were 16.9 million persons, with the age distribution being 23%, 25%, 35%, 13% and 4%, respectively. In the Netherlands, 137,561 deaths occurred in 1996, and 147,134 deaths in 2015. The age-standardized counts—further termed number of deaths—are available in an additional file (see Table S1).

We assessed time-trends in CODs by applying two complementary methods: identification of gradual long-term trends by means of polynomial models, and identification of sudden year-by-year changes by means of outlier detection.

In more detail, we assessed how many CODs (at the ICD-10 three-position level) would demonstrate a statistically significant change in mortality over a 20-year period. Possible trends were assessed by applying four different linear regression models, with polynomial terms of year added as independent covariates. We used orthogonal polynomials that account for multicollinearity of the polynomial components [6]. We applied the constant, the linear, the quadratic and the cubic model and evaluated them with the following hierarchical approach. Firstly, all four models were fitted, and we used the lowest corrected Akaike Information Criterion (AICc) to select the best model. We used the AICc as it performs better than the AIC for small sample sizes [7]. Secondly, the best model for each COD was compared with the constant model using the F-test, at a significance level of α = 0.05. If the best model performed better than the constant model with statistical significance, it remained the final best model. Otherwise, the constant model became the final best model for this COD.

In the development of the four polynomial models, a bivariate step parameter (break point) for deaths before or during/after 2013 was introduced, to allow for the possible effect of the introduction of automated coding of mortality data, which replaced the manual method of coding in the Netherlands in 2013 [8], as well as the implementation of the cumulative WHO updates to ICD-10, for the period 1996–2013 [9].

The association between COD size and likelihood of fitting a long-term trend (linear, quadratic, cubic) was analyzed with multinomial logistic regression, taking the logarithm of the mean number of deaths for the COD size, since its distribution was highly skewed.

Next, we assessed the number of CODs with significant sudden changes across the monitoring period. For each COD, we aimed to detect years with extreme observations in the number of deaths, which were defined as residuals of the final best model, at the significance level (alpha) of 0.01 and 0.001. These cases were identified using the outlierTest function from the “car” package in R software, with a cut-off of 0.01 and 0.001, respectively. The Bonferroni p-values were obtained assuming a *t*-distribution with degrees of freedom (df) equal to the residual df for the model minus one. [10].

In order to illustrate how the use of condensed lists of ICD-10 codes can lead to other selections when monitoring causes of death, we repeated the analysis of long-term trends with ICD-10 three position codes, aggregated as “chapters” or “blocks” of conditions [1]. The ICD-10 consists of 22 chapters, 19 of which are used for coding the underlying cause of death, and each chapter consists of one or more blocks of three position codes. For example, Chapter IX (Diseases of the circulatory system) includes a block like I20–I25 (Ischaemic Heart disease), which includes diseases like I21 (Acute Myocardial Infarction).

In the analysis of long-term trends, we used linear regression models, assuming normal error distribution, as we were primarily concerned with changes in absolute terms. Yet, statistical theory says that numbers of deaths are Poisson distributed, and that regression models with a log–linear link function would be more appropriate. However, the approximation of a Poisson by a normal error distribution is said to be adequate if the mean number of observations is about five or more. Moreover, in sensitivity analysis, we found that the main findings would not substantially change if we were to use Poisson regression instead of normal regression analysis (results not shown). 

All analyses were conducted with R software (3.3.1 version) [11].

No ethics approval or consent to participate was necessary, as we used publically available population data.

## 3. Results

For a substantial amount of CODs (216, 34.5%), we could not detect a long-term (linear, quadratic, cubic) trend (Table 1). For the remaining 409 causes, an annual trend was detected, following a linear (211), quadratic (126) or cubic (72) model. 

The descriptive statistics of the association between the probability of detecting an annual trend in mortality and the size of cause of death are given in Table 1. As expected, polynomial models of higher order were more often selected when the median number of deaths increased. The median number of deaths in the group of causes that were best described by the constant model, was 9.5 (20.4 for linear model, 38.1 for quadratic model and 56.2 for cubic model). Among all 625 CODs, a trend could be detected in 65.5% of cases (33.8% for linear model, 20.2% for quadratic model and 11.5% for cubic model). 

The association between COD size and selected polynomial degree was significant at α = 0.001. For every 10% increase in the mean number of deaths, there was a 3% increase in the odds of having the linear, as opposed to the constant, model (OR: 1.03, 95%CI:1.02;1.05). Similarly, the odds of having the quadratic or the cubic model compared to the constant model increased by 6% (OR: 1.06, 95%CI: 1.04;1.07) and 8% (OR: 1.08, 95%CI: 1.06;1.09), respectively. When the mean number of deaths was doubled, the correspondent changes in the odds were 27%, 49% and 71%.

**Figure 1:**
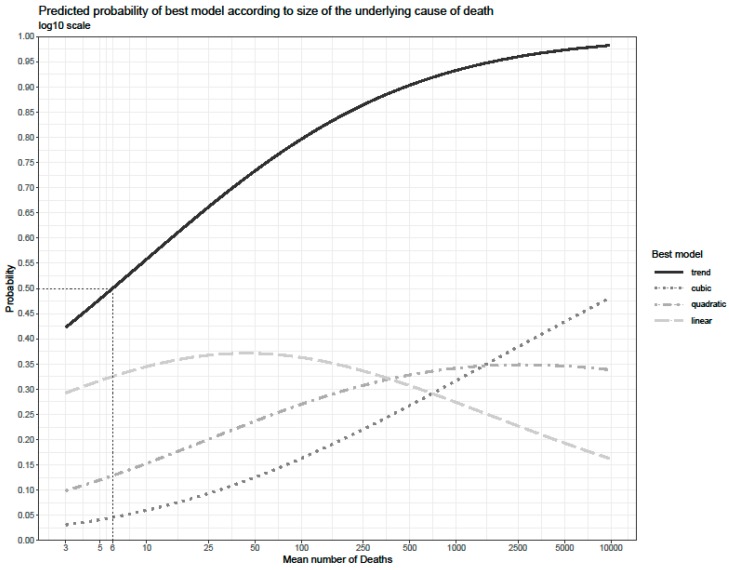
Predicted probability of detecting a long-term trend, according to size of the underlying cause of death.

Figure 1 gives the estimated probability of a COD having any long-term trend, defined as either a linear, a quadratic or a cubic final best model. The probability of having any sort of long-term trend (represented by the continuous line at the top of the graph) increased from 50% at a mean number of six deaths, to about 65% at 22 deaths and 75% at 60 deaths. The probability of having a linear trend (represented by the dashed line) increased as the mean number of deaths rose, reaching a peak at about 50 deaths. The subsequent decline is due to the increased probability of observing a quadratic trend (which reaches a peak at about 500 deaths) and a cubic trend (which steadily increased). Simultaneously, this reflects a shift towards higher order trends with increasing COD size.

When we move from the individual three-position ICD-10 codes towards aggregated lists of ICD-10 chapters, or blocks, for the study of long-term trends of causes of death, the number of CODs with a detectable trend decreased considerably, from 409 three-position codes to 121 blocks and 12 chapters, respectively (Table 2). 

Table S2 gives detailed information for each individual COD with a detectable trend. A great variety of trends was observed. The 211 CODs with a linear trend showed either a monotonous increase or a monotonous decrease. Of the 72 CODs with a cubic model, some showed an initial decrease, interrupted by a stagnation or increase, and followed by a second decrease, while other CODs followed the opposite pattern (initial increase, interrupted by stagnation or decrease, and subsequently a new increase). Only a few CODs showed no long-term trend, but instead had a few peak years with high mortality.

Regarding the investigation of sudden changes in causes of death, in total, 43 out of the 625 causes of death had an outlier observation year in the 20-year period at the 0.01 alpha level (not reported here). For the outlier observations at the 0.001 alpha level (Table 3), there were 16 CODs, with 14 having extremely high values, and two having an extremely low value, in one single year. Of these 16 CODs, nine were best described by the constant model, whereas a long-term trend could be identified for seven CODs.

## 4. Discussion

The objective of this paper was to determine how many of the CODs, at the ICD-10 three-position level, reveal an annual trend or a short-term fluctuation in the Netherlands, over a period of 20 years. The study outcomes could offer a criterion for deciding which ICD-10 codes should be distinguished when describing trends in a wide range of causes of death. This study could be particularly relevant in view of the forthcoming release of the ICD-11 version, which may increase the number of possible COD codes.

A long-term trend could be identified for about two thirds of the CODs with at least three annual deaths on average, and no long-term trend for the remaining one third. The probability of detecting a time trend increased from 50%, at a mean annual number of six deaths, to about 65% at 22 deaths, and 75% at 60 deaths. An exceptionally high or low number of deaths in one year could be demonstrated for only few CODs.

### 4.1. Evaluation of Data and Methods

The coding of causes of death at Statistics Netherlands may have affected observed trends for three different reasons: the delayed consequences of the introduction of the ICD-10, the change to automated coding, and other incidental changes. 

Firstly, the introduction of the new ICD-10 classification version, in 1996, in the Netherlands, which replaced the ICD9, may have been followed by temporal re-adjustments in the coding of COD during the first few years after 1996, such as the HIV codes (B20, B24), which were introduced for the first time in the ICD-10. Other examples are unspecified renal failure (N19), and respiratory distress of newborn (P22). Our finding that multiple valve diseases (I08) had an extremely high number of deaths in 1996 was already noted in a study aimed at detecting the effects of changes in data production during the period 1970–2006 [12].

Secondly, in our study we accounted, to a large extent, for the introduction of the automated COD coding and related changes in 2013. Switching from manual to automated coding can result in significant changes in cause-specific mortality rates [13,14,15]. In further analyses, we found that our results would be substantially affected if we omitted the 2013 step parameter and, thus, ignored the switch to automated coding. This would have decreased the proportion of CODs without a detectable long-term trend (from 34.5% to 26.6%), or with a linear model (from 33.8% to 27.8%), and increased the proportion of CODs with a quadratic model (from 20.2% to 23.5%), and cubic model (from 11.5% to 22.1%). While the inclusion of the step parameter was intended to prevent from identifying spurious time trends, it implements a conservative approach, that may come at the price of masking some real trends.

Thirdly, several incidental changes in the coding of COD may underlie some of the large short-term fluctuations that we observed. Other degenerative diseases of the nervous system, not elsewhere classified (G31), showed a 6.5-fold increase in 2015 as compared to 2013, most likely because of the implementation of a WHO ICD-10 update [9]. Changes in the coding process in Statistics Netherlands occurred in the period 1999–2002, when less resources for quality control were available. This may have contributed to changes in the coding of conditions, such as depressive episode (F32), as well as codes described as “other” or “unspecified”, such as other diseases of anus and rectum (K62), other and unspecified disorders of circulatory system (I99) and unspecified jaundice (R17). 

There are some limitations in the methodology used in our study. Firstly, we focused on codes at the ICD-10 three-position level and did not examine the more detailed codes at the ICD-10 four-position level. Although this could increase the number of the CODs with a detectable long-term trend, the sporadic occurrence of most four-position codes would add the potential for redundant analyses. Secondly, we did not investigate changes within individual years. For some CODs, such as contagious diseases with epidemic outbreaks, it may be more important to monitor day-to-day or week-to-week changes, rather than year-to-year trends. Thirdly, we included CODs with three or four deaths annually, even though it is uncertain whether deaths were normally distributed, as we assumed by applying normal regression. In a sensitivity analysis, we restricted the regression analysis to CODs with five or more deaths annually. We found that the probability of detecting a long-term trend was 50% at five mean annual deaths, 65% at 20 deaths and 75% at 60 deaths—results that are very close to our main findings. 

### 4.2. Interpretation of Trends

While we described long-term trends in mortality, we did not aim to explain the trends that were observed. In general, understanding long-term changes in mortality requires consideration of the incidence and case-fatality of a disease, the latter being largely determined by changes in medical practices and technologies, and in the provision of and access to health care [16,17]. Generally, relevant changes occur slowly and with delayed effects. Without any additional external information, such as personal data obtained from record linkages, it is difficult to attribute these long-term changes to specific factors, such as changes in treatments or risk factor prevalence [18]. One factor, the ageing of the Dutch population, was controlled by using the age-standardized death counts.

Some of the observed short-term fluctuations may reflect real changes in the occurrence of specific diseases or injuries in the Netherlands. One example is influenza due to an identified influenza virus (J10) that had an extremely low observation in 2014, which was not attributable to an exchange with J11 (influenza, virus not identified). Real changes in external causes of death are exemplified by operations of war (Y36), which increased in 2014, due to the MH17 flight crash.

Further research might show whether, and how, our results are cross-nationally generalizable. Some results are not directly generalizable to other countries, as the Netherlands has a medium-sized population (mean population during the study period was 16.3 million persons), and the proportion of CODs with a detectable trend is likely to be higher (lower) in countries with a substantially larger (smaller) population. However, the fundamental relationship between COD size and the likelihood of detecting a long-term trend might be applicable to other countries, and could be the focus of international comparative studies.

## 5. Conclusions

This study demonstrates, for the Dutch population, that a long-term mortality trend can be identified for at least 409 CODs, with a curved instead of linear trend in many cases. The size of a COD is an important predictor of the probability of detecting a long-term trend. Year-to-year mortality fluctuations could be demonstrated in fewer CODs and may result from problems with the coding of CODs.

In the context of the ICD11 release, with a large increase in the number of possible COD codes, and a decrease in the average number of deaths per COD, this study provides new evidence to guide the level of COD coding and reporting that should reasonably be recommended. Such recommendations are particularly important to national statistical offices, as they have to decide what level of detail to include when they publish annual cause-specific mortality data. Both producers of these statistics and users of the data benefit from realizing that, despite the large number of ICD codes that could be distinguished, the number of CODs for which a trend can be detected is limited. At the same time, it is important to realize that there is a reasonable likelihood of detecting a long-term trend, even for CODs with only three annual deaths. Our results therefore suggest the selection of a broad range of causes of death, preferably defined in terms of a minimum annual number of deaths, rather than condensed lists of CODs, in order to not overlook CODs with a significant long-term trend.

## Figures and Tables

**Table 1:** Distribution of the Causes of Death (ICD-10 three-position), according to the final best model, stratified by their size.

	Final Best Model				
	Constant (No Detectable Trend)	Linear	Quadratic	Cubic	Total
Cause of Death Size ^a^	Number of Causes of Death (% of all Causes of Death with that Size)				
[3,5)	55 (59.2)	27 (29.0)	8 (8.6)	3 (3.2)	93 (100.0)
[5,15)	78 (45.1)	64 (37.0)	24 (13.9)	7 (4.0)	173 (100.0)
[15,30)	32 (29.4)	38 (34.9)	23 (21.1)	16 (14.7)	109 (100.0)
[30,100)	35 (27.8)	44 (34.9)	31 (24.6)	16 (12.7)	126 (100.0)
[100,9750)	16 (13.0)	38 (30.6)	40 (32.5)	30 (24.4)	124 (100.0)
Total	216 (34.5)	211 (33.8)	126 (20.2)	72 (11.5)	625 (100.0)
Median number of deaths [IQR] ^b^					
	9.5 [4.9–27.4]	20.4 [7.7–56.7]	38.1 [14.8–162.4]	56.2 [22.0–479.2]	20.7 [7.6–71.8]

^a^ [*i*,*j*) is an interval notation for all values between *i* (included) up to *j* (not included). ^b^ IQR: interquartile range.

**Table 2:** Distribution of the final best model of ICD-10 Causes of Death at different levels of aggregation.

Final Best Model	Three-Position Codes	Blocks	Chapters
Constant (no detectable trend)	216	41	5
Any detectable trend	409	121	12
linear	211	56	6
quadratic	126	42	4
cubic	72	23	2
Total	625	162	17

Trend: the composite of linear, quadratic and cubic final best models.

**Table 3:** Causes of death (ICD-10 three-position) with a detectable mortality fluctuation at the alpha 0.001 level.

ICD-10 Code	ICD-10 Code Label	Observation Year	Deaths in Observation Year (O)	Mean Deaths in Other Years (E)	Ratio O/E	Final Best Model
B24	Unspecified human immunodeficiency virus [HIV] disease	1996	97.9	18.7	5.24	quadratic
B94	Sequelae of other and unspecified infectious and parasitic diseases	2013	2.4	3.1	0.77	constant
F32	Depressive episode	2001	54.3	28.3	1.92	constant
G31	Other degenerative diseases of nervous system, not elsewhere classified	2015	408.2	90.1	4.53	constant
I08	Multiple valve diseases	1996	58.6	19.5	3.01	cubic
I99	Other and unspecified disorders of circulatory system	2002	75.1	25.7	2.92	quadratic
J09	Influenza due to certain identified influenza virus	2009	31.7	1.6	19.81	linear
J10	Influenza due to identified influenza virus	2014	9.3 ^a^	9.0	1.03	constant
K62	Other diseases of anus and rectum	2001	40.8	11.5	3.55	constant
K66	Other disorders of peritoneum	2002	14.8	3.2	4.63	constant
N19	Unspecified renal failure	1996	696.3	361.6	1.93	quadratic
Q27	Other congenital malformations of peripheral vascular system	2005	16	2.6	6.15	constant
R17	Unspecified jaundice	1999	28.9	12.5	2.31	constant
V45	Car occupant injured in collision with railway train or railway vehicle	1999	29.2	7.4	3.95	linear
Y36	Operations of war	2014	183.8	1.0	183.8	constant
P22	Respiratory distress of newborn	1996	52.4	7.9	6.63	cubic

^a^ Cause of death J10 has substantially decreased in number of deaths in 2014, compared to 2013 and 2015 (i.e., years after the change to automatic coding), but has a similar number of deaths compared to years in the period 1996–2012.

## Supplementary Materials

The following are available online at https://www.mdpi.com/1660-4601/16/21/4150/s1, Table S1: Age-standardized count of deaths for the three position ICD-10 codes reported in the Netherlands, for the period 1996–2015. Table S2: Detailed long-term trend patterns for the 625 three position ICD-10 codes with at least three average annual deaths as individual causes, or aggregated per block and chapter.
